# Case-Crossover Method with a Short Time-Window

**DOI:** 10.3390/ijerph17010202

**Published:** 2019-12-27

**Authors:** Mieczysław Szyszkowicz

**Affiliations:** Environmental Health Science and Research Bureau, Health Canada, Ottawa, ON K1A 0K9, Canada; mietek.szyszkowicz@canada.ca

**Keywords:** air pollution, case-crossover, cluster, concentration, counts, time-series

## Abstract

Numerous epidemiological studies have shown associations between short-term ambient air pollution exposure and various health problems. The time-stratified case-crossover design is a popular technique for estimating these associations. In the standard approach, the case-crossover model is realized by using a conditional logistic regression on data that are interpreted as a set of cases (i.e., individual health events) and controls. In statistical calculations, for each case record, three or four corresponding control records are considered. Here, the case-crossover model is realized as a conditional Poisson regression on counts with stratum indicators. Such an approach enables the reduction of the number of data records that are used in the numerical calculations. In this presentation, the method used analyzes daily counts on the shortest possible time-window, which is composed of two consecutive days. The proposed technique is positively tested on four challenging simulated datasets, for which classical time-series methods fail. The methodology presented here also suggests that the length of exposure (i.e., size of the time-window) may be associated with the severity of health conditions.

## 1. Introduction

The case-crossover (CC) study design is an approach where each individual case serves as its own control [1]. The CC method is often and widely used in environmental epidemiology to estimate the risk of a health event related to short-term (acute) exposure to ambient air pollution. Specifically, this method is usually applied for investigating the transient effects of an intermittent exposure on the onset of acute events.

The standard CC technique usually uses one month as its time-window. The strategy to determine for a case corresponding control(s) is usually based on a time-stratified approach [2]. In the CC method, the day of week is not modeled but is adjusted by the realized design. For the specific case day, the potential controls are generated as multiples of seven from the case day in both directions, pre- and post-event day (±w x 7, where w=1, 2, 3, 4). The control days are then chosen among the proposed days that belong to the same one common month [2]. This process results in three or four control days, depending on the length of a month (28, 29, 30, or 31 days) and the day of the week. For example, according to this scheme, for the case of the day of 9 November 2019, there are four control days: 2, 16, 23, and 30 November. In such scenarios, the cases (events) and controls are still related to time. The pattern of sequences of case-control(s) varies in time. We may have various configurations of pre- and post-event control days; for example, one pre and three post (see the example with the case day of 9 November) or two pre and two post. In the original presentation of the CC method [1], one control was defined, and the control always occurred seven days before the time event. Such a fixed-time configuration of case-control relations usually results in bias from time trends in exposure prior to the occurrence of an adverse event.

To reduce the bias in the CC method, a few authors [3,4,5] have presented modifications to the CC technique and have used daily counts rather than individual health events (i.e., case-control relations). Their proposed methods are realized by using clusters (strata) that control for time by the applied hierarchical structures based on the calendar relations <year: month: day of week>. Daily counts are grouped and considered on the defined strata. According to the assumed convention, daily counts on 2, 9, 16, 23, and 30 November all belong to the same stratum that is determined by the structure <year = 2019: month = November: day of week = Saturday>. It is also good to note that, in this approach, the corresponding regression is not affected by time (pre- and post-event scenarios) in the time-window used; rather, it is affected by the concentration levels of the air pollutant considered. Time is controlled by the constructed strata, and it is eliminated from the regression. Thus, the “control is always before case” effect or similar effects, which are usually seen in the CC method and generate bias, are lessened. It is possible to construct strata of various sizes. Among the proposed ones is the CC method that uses a two week time-window, i.e., with the strata of the form <year: two week: day of week>. These strata have only two days [3,5]. This is different from the originally proposed CC method that only uses one control [1] for each health case. In this situation, the time duration is also two weeks but is interpreted differently. Say that we consider the daily counts on two consecutive Mondays, and the method is realized with the use of the stratum <year: two week: day of week>; in the originally presented CC method, for the case of a Monday, its control is the Monday one week before. In both approaches, two data points are enough to determine a slope (beta) between the air pollution concentration and health outcomes. The authors of [6] proposed another method to reduce bias in CC methods.

In the time-series study considered here, a few modifications and adaptations of the CC method are proposed and used to control for the bias related to the size of the applied time-window. In one of the time-series study papers by Burr et al., it was stated [7]: “We additionally showed that the use of 6 df/year for a smooth function of time is not, in general, sufficient to protect estimates from seasonal variation. The use of natural cubic regression splines at higher df/year (e.g., 12) will protect slightly better against seasonal variation than 6 df/year but still suffers from the poor concentration properties of its family.”

This issue was among the reasons for doing the present study. As the authors showed in their publication [7], a classical time-series methodology did not work for their challenging simulated data. As a solution, they proposed alternative smooth functions. This work presents a simple and effective solution to the bias correction problem by applying a CC method technique that is realized over a short time window.

## 2. Materials and Methods 

This study was conducted by using the simulated data (four sets: Sim1–Sim4) and mortality data from Chicago, IL, USA [7]. The original Chicago mortality data were provided by the NMMAPSdata database [8]. Here, the attention was restricted to two air pollutants (trimmed mean daily coarse particulate matter and trimmed mean daily ozone). The mortality data contained daily all-cause mortality counts (death) and daily cardio-vascular mortality counts (CVD), for the period of 1987–2000. These databases are exactly the same as in the publication for the time-series study [7], and all their details are presented in the original time-series work. The simulated data and code (in *R*, [9]) that were used are available at https://github.com/szyszkowiczm/Data2D. The Supplementary Material associated with the publication of Burr et al. [7] contains the details of the simulated data. In many calculations of short-term risk related to air pollution, smooth functions of time are realized as fixed-df cubic regression splines. In this presentation the constructed strata were used to control time.

**Figure 1:**
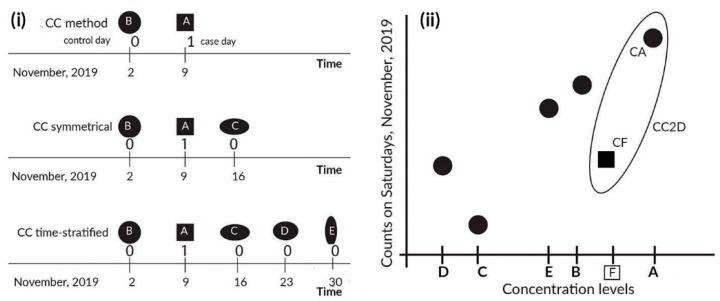
Left panel (**i**) shows variants of the case-crossover (CC) methods, with three control schemes {−7,0}, {−7,0,7}, and {−7,0,7,14,21}, where 0 is an event day, −7 is one week before event, 7 is one week after event, etc. Thus, {−7,0} results in two days; 9 November as an event day and 2 November as a control day. Right panel (**ii**) shows the points used in the realization of the time-stratified CC methods with counts (the CCM method, which has hierarchical clusters of the form <year: month: day of week>). The exposure levels are A–E and can be lagged. The CC2D method (the method that uses the hierarchical calendar structure of <year: two days>) for just one pair—(A, CA), (F, CF)—is shown in the ellipsis; exposures A and F result in counts CA and CF, respectively.

In this study, we employed a CC method that uses the hierarchical calendar structure of <year: two days>, noted here symbolically as <year: 2D>; this version of the method is called the CC2D method. For each individual year, consecutive days were grouped in pairs (2D), specifically (1, 2), (3, 4), (5, 6), … etc. For example, the pair of (1, 2) represents the first and second day of January and the pair of (3, 4) represents the next two days of this month. In the constructed models, we adjusted for the day of week. This was the shortest possible time-window in the CC method to analyze daily counts. In this situation, the stratum had two days. Figure 1 illustrates two approaches: the case-crossover realized with events and with counts.

Here, we realized conditional Poisson regression as an alternative approach to the conditional logistic regression technique used in the standard CC model. The package “gnm” was used to specify and fit generalized nonlinear models to the defined stratum (here, <year:2D>).

The following model in the *R* software [9] was built for the case of the simulated data (four different categories: Sim1–Sim4, with 250 samples each)

modM <- gnm(y ~x + dowf, data = data,
family = gaussian, eliminate = factor(stratum)),

where y is response (health outcome), x is exposure (air pollution concentration), and dowf is the day of week as a factor. The stratum (cluster) is defined as data$stratum <- as.factor(data$year:data$2D). In the model, there are hierarchical clusters with two structure levels: two days embedded in a year and 183 clusters per year. In this study, the simulated data used were artificial and represented 10 years (3650 days) of time-series sequences (exposure–response); thus, each year was a series of 365 days. The simulation data were not counts and were generated by the authors [7] (Burr et al., 2015) to investigate their technique and its properties. Simulation 1 consisted of two periodicities at periods 183 and 75 days. Simulation 2 extended Simulation 1 by including three additional signals. The signals were added at periods of 68 and 105 days. Simulation 3 used a similar signal structure as Simulation 2 but changed the background noise. Simulation 4 was based on the same signal structure as Simulation 3 but was scaled by a factor of the two background noises [7].

In addition, for comparison purposes, we also considered the following models; CC3D—similar to the CC2D model but with the stratum based on a three-day structure <year:3 days>; CC2W —with a 3-level stratum of the form <year: two week: day of week>; and CC2CW—with a 3-level stratum as in CC2W but “chained,’ as each day was used twice in the neighbor strata, e.g., (1, 2), (2, 3), and (3, 4). [5]. As the simulated data were challenging for the analysis, we realized these methods, CC2D, CC3D, CC2W, and CC2CW, to observe their performance.

Additionally, to analyze the mortality Chicago data, models with the hierarchical clusters of the form <year: month: day of week> (CCM) were also used. In the constructed models, family = Poisson was set in the *R* code, and this model also included ambient temperature. The temperature was represented in the form of natural splines with three degrees of freedom. In this situation, we had real epidemiological data.

## 3. Results

The simulated data posed rather difficult and challenging problems for the time-series approach [7]. The true value for the slope was one (beta = 1.0) for each simulated series (Sim1–Sim4). According to the authors of the original publication [7], the simulated data that used the time-series method, executed with natural cubic regression splines for time with 6 df/year, produced the following slope estimates: 0.284, 0.064, 0.139, and −0.177 (negative) for the data from Sim1 to Sim4, respectively. These were the average values of the estimated slopes with 250 samples for each simulation (as presented in Table 1 in the paper of Burr et al. [7]).

**Figure 2:**
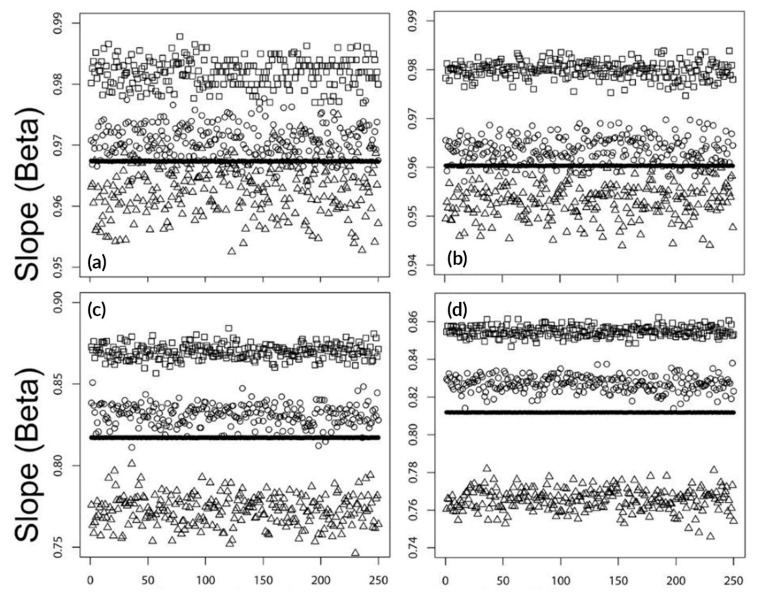
Estimated slopes (beta, true beta = 1.0) for four sets of simulation data (Sim1–Sim4) with 250 samples each. The panels illustrate the results for the following methods: (**a**) CC2D, (**b**) CC3D (similar to the CC2D model but with the stratum based on a three-day structure <year:3 days>), (**c**) CC2W (with a 3-level stratum of the form <year: two week: day of week>), and (**d**) CC2CW(with a 3-level stratum as in CC2W but “chained,’ as each day was used twice in the neighbor strata). The simulation data are identified by the following symbols: Sim1—square; Sim2—black circle (seen as a solid black line, as the values are almost identical); Sim3—circle; and Sim4—triangle.

**Figure 3:**
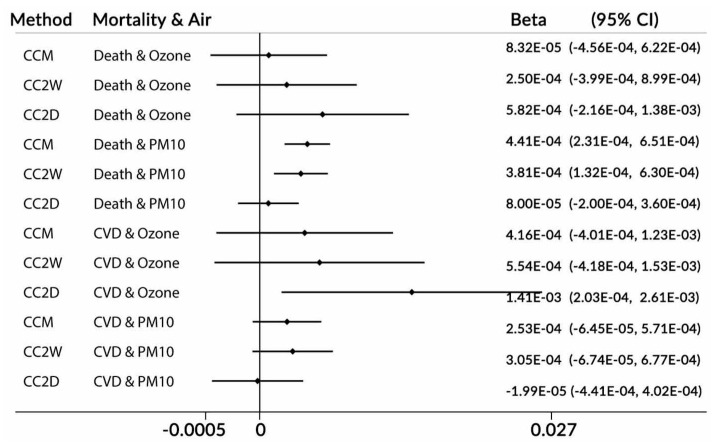
Estimated slopes (beta) for mortality data. Chicago, USA, 1987–2000. Note: CC—case-crossover method; M, 2W, and 2D—time-windows of one month, two weeks, and two days, respectively. CVD—cardio-vascular mortality; beta—slope; and CI—confidence interval.

The results obtained are summarized in Figure 2 and Figure 3. Table 1 and Table 2 present the numerical values generated for the simulated data. Figure 1 shows the estimated slope values for each series that used the simulated data (Sim1–Sim4). These series had 250 samples each (4 series x 250 samples), and each has 3650 days, i.e., 10 years. 

The figure illustrates the results for the four methods of CC2D, CC3D, CC2W, and CC2CW in panels (a)–(d), respectively. The CC method on the cluster <year:2D>, i.e., the CC2D method, have very accurate estimates of the slopes. The estimated average slopes from the simulations were 0.9819, 0.9674, 0.9707, and 0.9628, respectively (where the true beta = 1.0) with standard deviations 2.26E-03, 4.81E-05, 2.80E-03, and 4.31E-03, respectively. Table 1 summarizes the numerical results for the CC2D and CC3D methods. Table 2 shows the same statistics of the results from the CC2W and CC2CW methods. The average values of the estimated slopes (beta1–beta4 for simulations (1–4) are shown in bold. Their standard errors are listed under SE1–SE4 for the corresponding simulation data.

Figure 3 represents the results for the mortality data in Chicago and ambient air pollutants. These are real ambient ozone and PM10 (particulate matter with diameters of no greater than 10 microns) air pollution concentration data. Three forms of the CC model were applied to process these data: CCM (one-month time-window), CC2W (two week time-window), and CC2D (two day time-window). The intention was to compare the standard CC method (CCM) with two kinds of two-day methods (CC2W and CC2D). 

In the cases of the CCM and CC2W approaches, the day of week was adjusted by the design, as was explained previously, in the form of the clusters used. In this situation, the day of week was one of the three levels of the constructed hierarchical clusters.

## 4. Discussion

The presented statistical technique, the CC method with two days as the time-window (CC2D), was easy to implement and use in short-term air health effects studies. The proposed method worked very well with the simulated data used here for illustrative purposes. Since in this case we knew the true value of the slope (beta = 1), it was easy to judge and validate the obtained results.

As the results for the simulated data presented in Table 1 and Table 2 indicate, the CC2D method gave the most accurate estimate of the true slope (beta = 1.0) among the other applied methods: CC3D, CC2W, and CC2CW.

As was already mentioned, the CC2CW was realized by using the clusters in the form <year: chained 2 week: day of week>. Two weeks were chained according to the following construction: (first week, second week), (second week, third week), (third week, fourth week), etc., separately for each year. This approach almost doubled the number of observations and narrowed the corresponding confidence intervals. According to the results presented in Table 2, the estimated slopes were 0.8549, 0.8118, 0.8270, and 0.7656, and their estimated average standard errors were 0.0084, 0.0094, 0.0090, and 0.0138, for Sim1–Sim4, respectively. These standard errors were smaller than those obtained for the CC2W method, as they were 0.0120, 0.0132, 0.0126, and 0.0193, respectively.

In the case of the mortality data in Chicago, we do not know the true effects of the exposures on the daily mortality counts. In the models applied, we adjusted for the ambient temperature by using natural splines with three degrees of freedom. The CC2D method indicated a positive association between ozone and death, as well as a statistically significant association for CVD mortality. The same types of the associations were obtained by the authors in [7] (see Figure 5 in [7]). The results appear very reliable because the CVD counts were a subset of the death counts. In the case of particulate matter exposure, the CCM and CC2W methods indicated positive statistically significant associations for death, but they only indicated a positive non-significant association for CVD deaths. The same type of relations was reported by the authors in [7]. It is strange that the results were not the same nature for CVD deaths as they were for death. The CC2D method did not show the associations for CVD deaths, all deaths and coarse particulate matter concentrations. It is difficult to make conclusions here, as the methods we used were different in nature. The CCW and CC2W methods used the same approach as case-control relationships, while the CC2D method used counts and was closer to the time-series technique than to the case-crossover methodology. The CC2D method estimated the slopes by using two neighboring days, so it may have been more related to acute events (see Figure 1).

## 5. Conclusions

The presented CC2D method performed very well on the four simulated datasets. This simple technique enabled an accurate estimate of the slope (beta = 1). In addition, we have a few conclusions: (a) The Conditional Poisson model is a flexible and reliable alternative to the conditional logistic case cross-over model; (b) using counts reduces the number of records in the realization of the CC technique [10]; and (c) the strata applied to control time may have various sizes. We also conclude that it is reasonable to run the CC2D model, at least to verify the associations suggested by other approaches.

## Figures and Tables

**Table 1:** Estimated parameters obtained for the CC2D and CC3D methods.

	CC2D Method				CC3D Method			
**Parameter**	**Beta1**	**Beta2**	**Beta3**	**Beta4**	**Beta1**	**Beta2**	**Beta3**	**Beta4**
**Minimum**	0.9769	0.9672	0.9619	0.9525	0.9747	0.9602	0.9571	0.9440
**1stQuartile**	0.9801	0.9673	0.9688	0.9599	0.9789	0.9603	0.9620	0.9513
**Median**	0.9820	0.9674	0.9708	0.9630	0.9800	0.9603	0.9638	0.9536
**Mean**	0.9819	0.9674	0.9707	0.9628	0.9799	0.9603	0.9637	0.9534
**3rdQuartile**	0.9835	0.9674	0.9726	0.9657	0.9810	0.9604	0.9655	0.9557
**Maximum**	0.9878	0.9675	0.9777	0.9750	0.9839	0.9605	0.9697	0.9621
**Parameter**	**SE1**	**SE2**	**SE3**	**SE4**	**SE1**	**SE2**	**SE3**	**SE4**
**Minimum**	0.0029	0.0051	0.0043	0.0062	0.0029	0.0048	0.0042	0.0062
**1stQuartile**	0.0030	0.0051	0.0044	0.0065	0.0030	0.0048	0.0043	0.0064
**Median**	0.0031	0.0051	0.0045	0.0066	0.0030	0.0048	0.0043	0.0065
**Mean**	0.0031	0.0051	0.0045	0.0066	0.0030	0.0048	0.0044	0.0065
**3rdQuartile**	0.0031	0.0051	0.0046	0.0066	0.0030	0.0048	0.0044	0.0065
**Maximum**	0.0033	0.0051	0.0048	0.0068	0.0032	0.0048	0.0046	0.0068

**Table 2:** Estimated parameters obtained for the CC2W and CC2CW methods.

	CC2W Method				CC2CW Method			
**Parameter**	**Beta1**	**Beta2**	**Beta3**	**Beta4**	**Beta1**	**Beta2**	**Beta3**	**Beta4**
**Minimum**	0.8565	0.8169	0.8112	0.7463	0.8469	0.8117	0.8138	0.7458
**1stQuartile**	0.8669	0.8170	0.8271	0.7664	0.8532	0.8118	0.8245	0.7617
**Median**	0.8698	0.8170	0.8318	0.7732	0.8547	0.8118	0.8271	0.7658
**Mean**	0.8699	0.8170	0.8311	0.7727	0.8549	0.8118	0.827	0.7656
**3rdQuartile**	0.8725	0.8171	0.8355	0.7789	0.8568	0.8118	0.8298	0.7691
**Maximum**	0.8842	0.8171	0.8509	0.8010	0.8623	0.8119	0.838	0.7819
**Parameter**	**SE1**	**SE2**	**SE3**	**SE4**	**SE1**	**SE2**	**SE3**	**SE4**
**Minimum**	0.0117	0.0132	0.0122	0.0186	0.0083	0.0094	0.0087	0.0133
**1stQuartile**	0.0119	0.0132	0.0125	0.0192	0.0084	0.0094	0.0089	0.0137
**Median**	0.0120	0.0132	0.0126	0.0193	0.0084	0.0094	0.0090	0.0138
**Mean**	0.0120	0.0132	0.0126	0.0193	0.0084	0.0094	0.0090	0.0138
**3rdQuartile**	0.0121	0.0132	0.0127	0.0195	0.0085	0.0094	0.0090	0.0138
**Maximum**	0.0123	0.0132	0.0130	0.0201	0.0087	0.0094	0.0093	0.0141

## Supplementary Materials

The following are available online at https://github.com/szyszkowiczm/Data2D—the simulated data and the corresponding codes in R.

## References

[1] Maclure M. (1991). The case-crossover design: A method for studying transient effects on the risk of acute events. Am. J. Epidemiol..

[2] Janes H., Sheppard L., Lumley T. (2005). Case-crossover analyses of air pollution exposure data: Referent selection strategies and their implications for bias. Epidemiology.

[3] Szyszkowicz M. (2006). Use of generalized linear mixed models to examine the association between air pollution and health outcomes. Int. J. Occup. Med. Environ. Health.

[4] Armstrong B.G., Gasparrini A., Tobias A. (2014). Conditional Poisson models: A flexible alternative to conditional logistic case cross-over analysis. BMC Med. Res. Methodol..

[5] Szyszkowicz M., Burr W.S. (2016). The use of chained two-point clusters for the examination of associations of air pollution with health conditions. Int. J. Occup. Med. Environ. Health.

[6] Wang X., Wang S., Kindzierski W. (2019). Eliminating systematic bias from case-crossover designs. Stat. Methods Med. Res..

[7] Burr W.S., Takahara G., Shin H.H. (2016). Bias correction in estimation of public health risk attributable to short-term air pollution exposure. Environmetrics.

[8] Peng R.D., Welty L.J. (2004). The NMMAPSdata package. R News.

[9] R Core Team (2016). R: A language and environment for statistical computing. R Foundation for Statistical Computing.

[10] Szyszkowicz M. (2019). The Air Quality Health Index and all emergency department visits. Environ. Sci. Pollut. Res. Int..

